# Supplementary material for: Signals from the head and germinative region differentially regulate regeneration competence of the tapeworm Hymenolepis diminuta
Source: Development. 2025 Oct 20;152(20):dev204781. doi: 10.1242/dev.204781 (PMC12582415; doi:10.1242/dev.204781)
Supplement: Supplementary information [file develop-152-204781-s1.pdf]

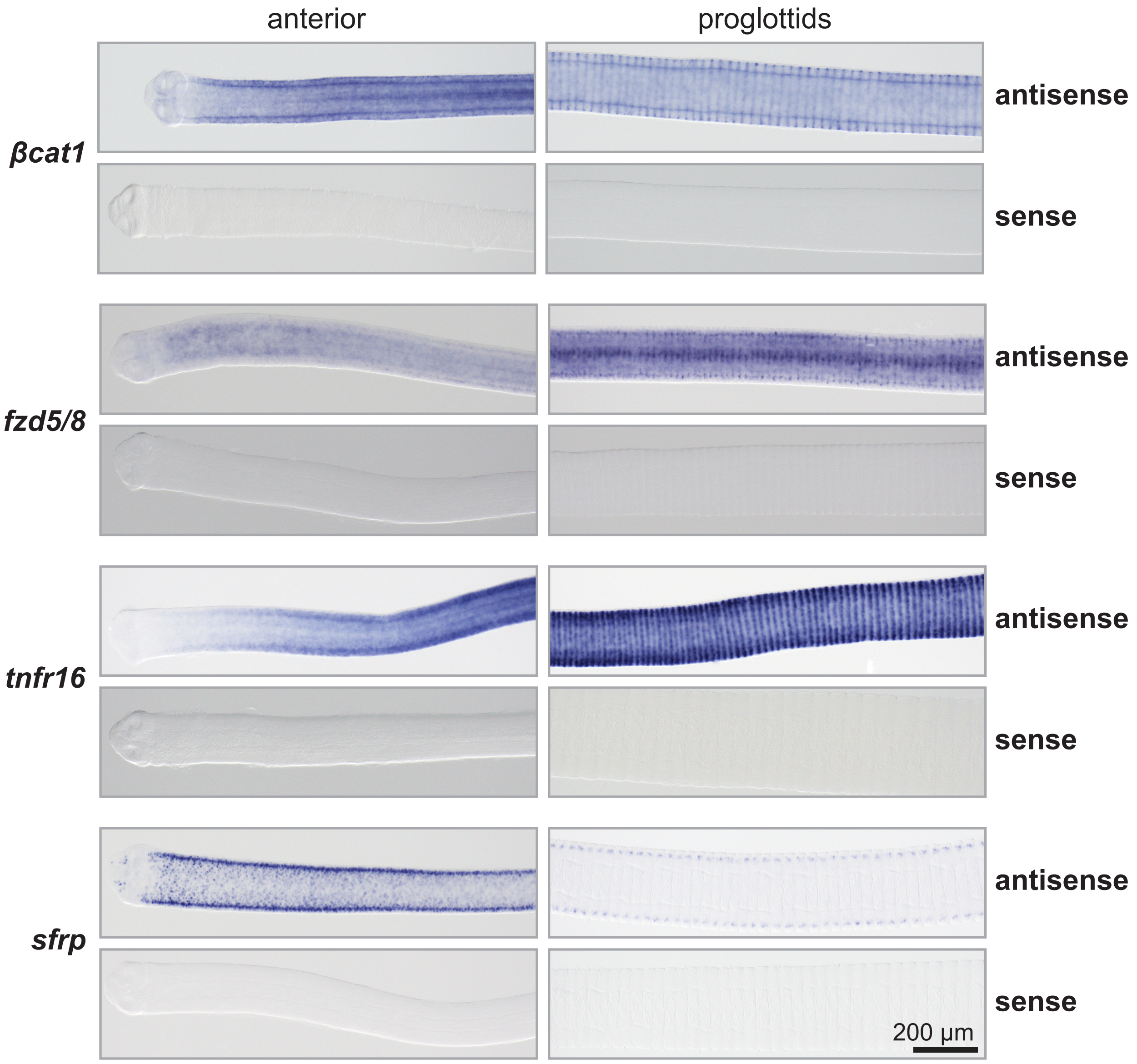

Fig. S1. Specificity of antisense riboprobes. WISH with antisense and sense riboprobes for transcripts in Fig. 1E.

Development • Supplementary information

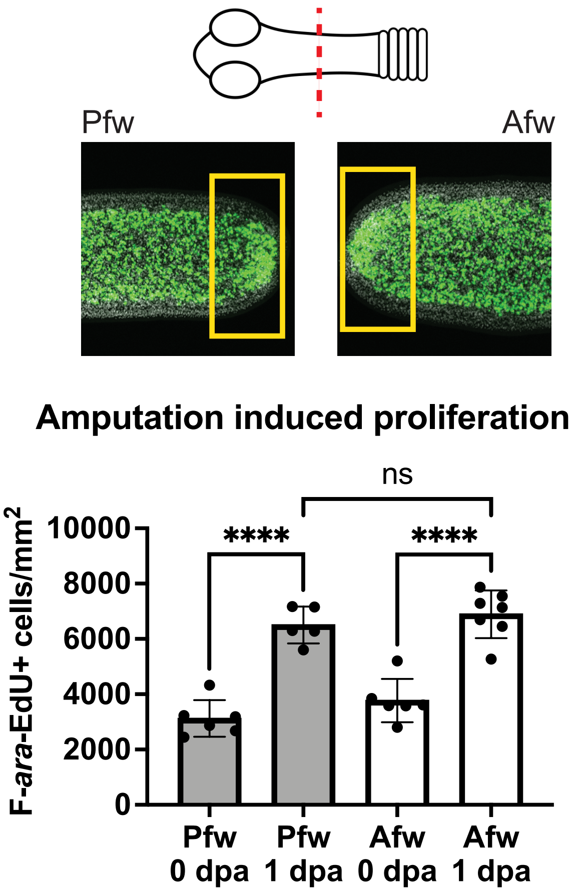

Fig. S2. Proliferation response at wound site. Worms were amputated at 0.5 mm within the GR. Proliferation density was quantified from a 100 μ m-wide box at the posterior-facing wound (Pfw) and anterior-facing wound (Afw) at 1 dpa after 1 hr pulse of F-*ara*-EdU. Quantification from one representative experiment, n= 6, 5, 6, 7; error bars= SD, one-way ANOVA with Tukey's multiple comparison test.

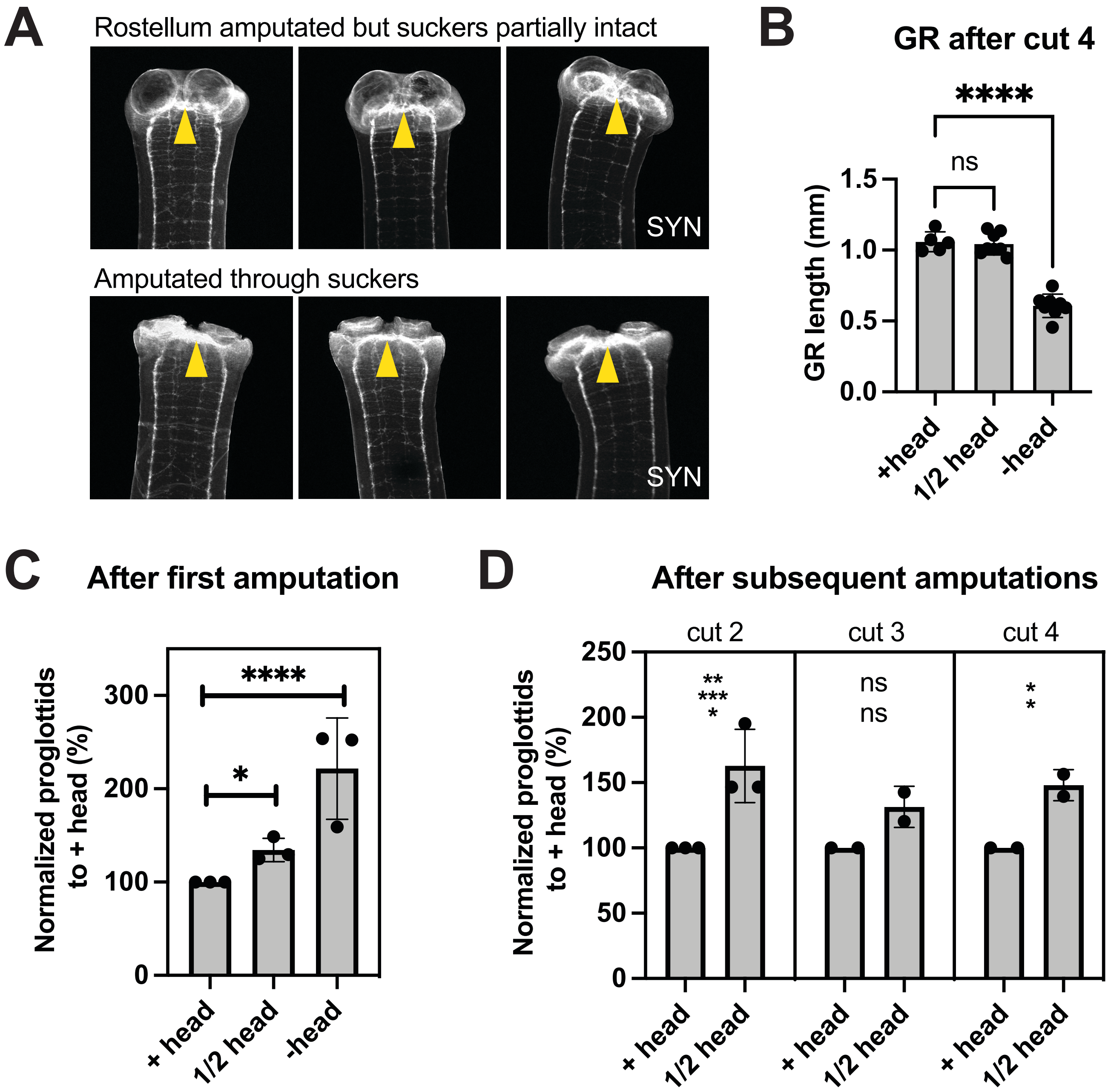

Fig. S3. How head tissues influence regeneration. (A) Widefield fluorescent images of ½ head fragments at 0 dpa stained with anti-SYN antibodies. Yellow arrowheads point to the brain/cephalic ganglia. (B) GR lengths at the end of a representative serial amputation experiment. n= 5, 8, 8; one-way ANOVA with Dunnett's multiple comparison test. (C) Quantification of proglottids regenerated after cut 1, normalized to +head means. N= 3; n= 27, 37, 26; statistical significance by one-way ANOVA and was the same for all 3 experiments. (D) Quantification of proglottids regenerated normalized to +head means. Cut 2: N= 3, n= 27, 34; Cut 3: N= 2, n= 11, 16; Cut 4: N= 2, n= 13, 15; t-tests. Error bars= SD.

Development • Supplementary information

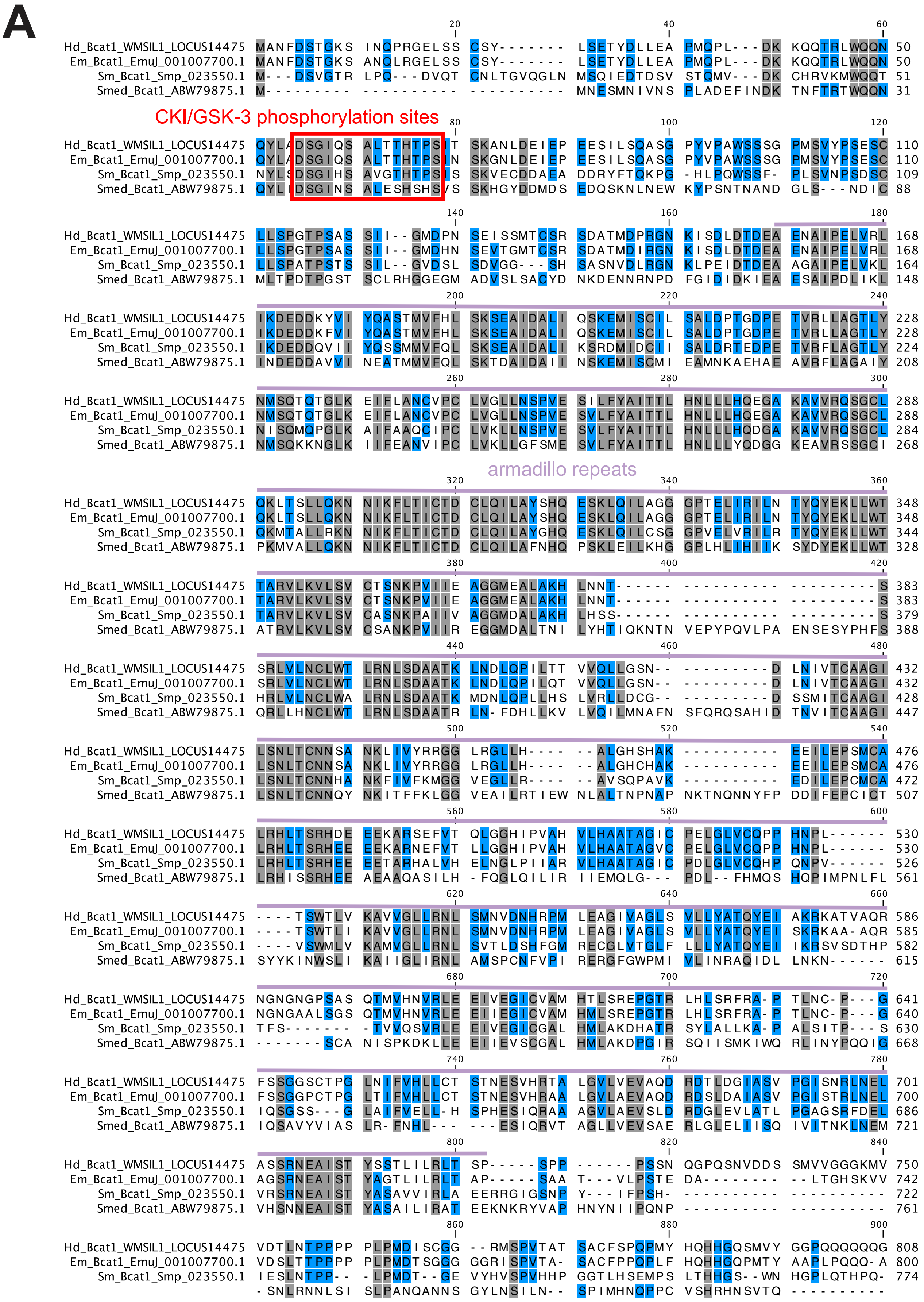
A
CKI/GSK-3 phosphorylation sites
armadillo repeats
Hd_Bcat1_WMSIL1_LOCUS14475
Em_Bcat1_EmuJ_001007700.1
Sm_Bcat1_Smp_023550.1
Smed_Bcat1_ABW79875.1

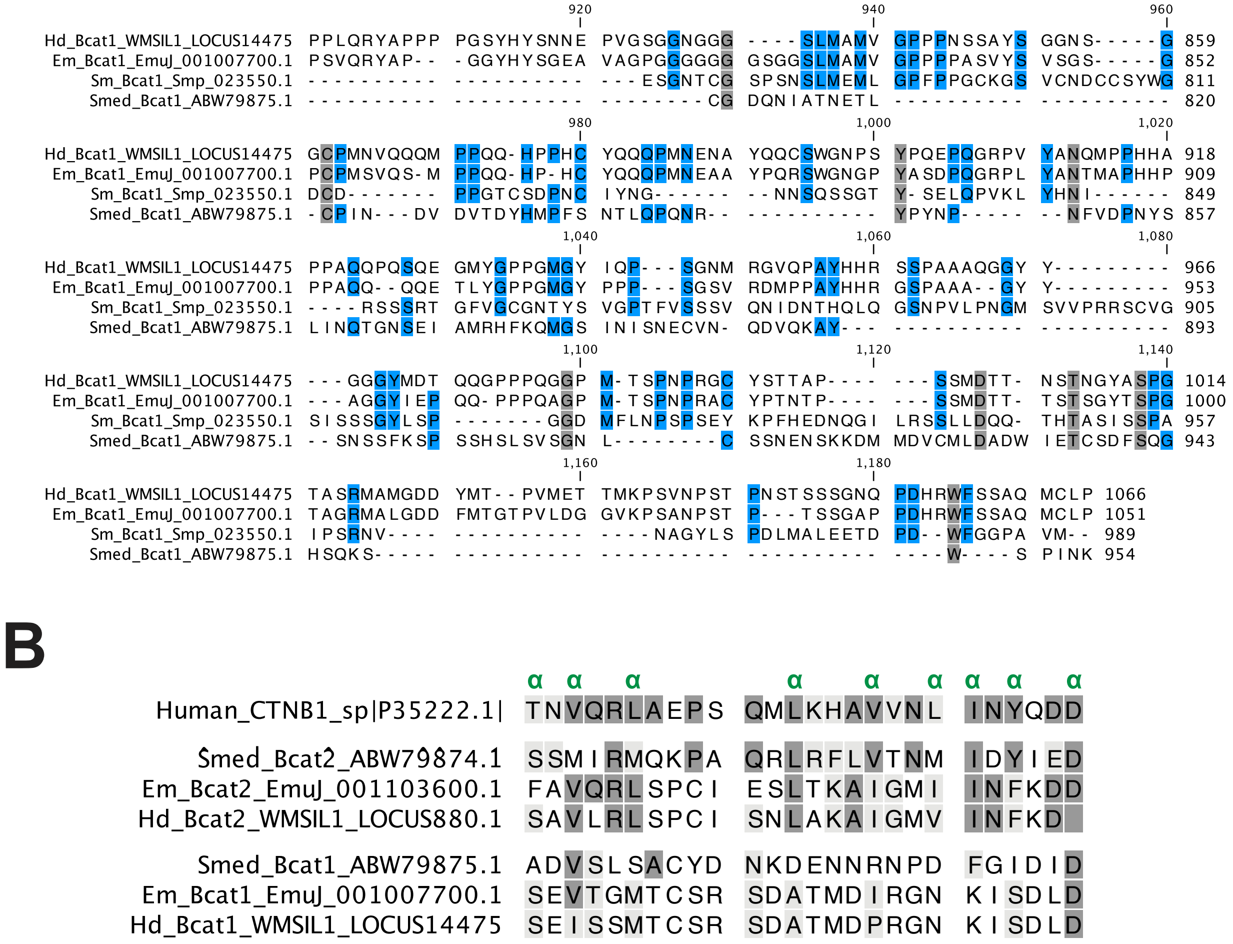

Fig. S4. Domain analysis of H. diminuta βCAT1. (A) Sequence alignment of *βCAT1* from four flatworms (Hd: *H. diminuta*, Em: *E. multilocularis*, Sm: *S. mansoni*, Smed: *S. mediterranea*). The conserved DSGxxSxxx[S/T]xxxS motif for CKI/GSK-phosphoryla-tion (red) is present as are armadillo repeats (lilac). The boundaries of both domains are shown according to *S. mediterranea* (Su et al., 2017). Shading indicates identical resi-dues between 4 species (gray) or 3 species (blue). The N- and C-termini are more diver-gent between all species. (B) Sequence alignment of 26 amino acids covering a putative α-catenin binding domain (Montagne et al., 2019). 10 critical residues for α-catenin bind-ing (Aberle et al., 1996; Pokutta and Weis, 2000) are marked by α (green). In reference to human CTNB1, dark gray shading highlights identical residues. Chemically similar residues marked in light gray for aromatic (F, Y, W, H), aliphatic (V, I, L), positively charged (R, K, H), negatively charged (D, E), polar but not charged (N, Q, T, S, Y, C) and hydrophobic (V, I, L, M, A, F, P, W, G) residues.

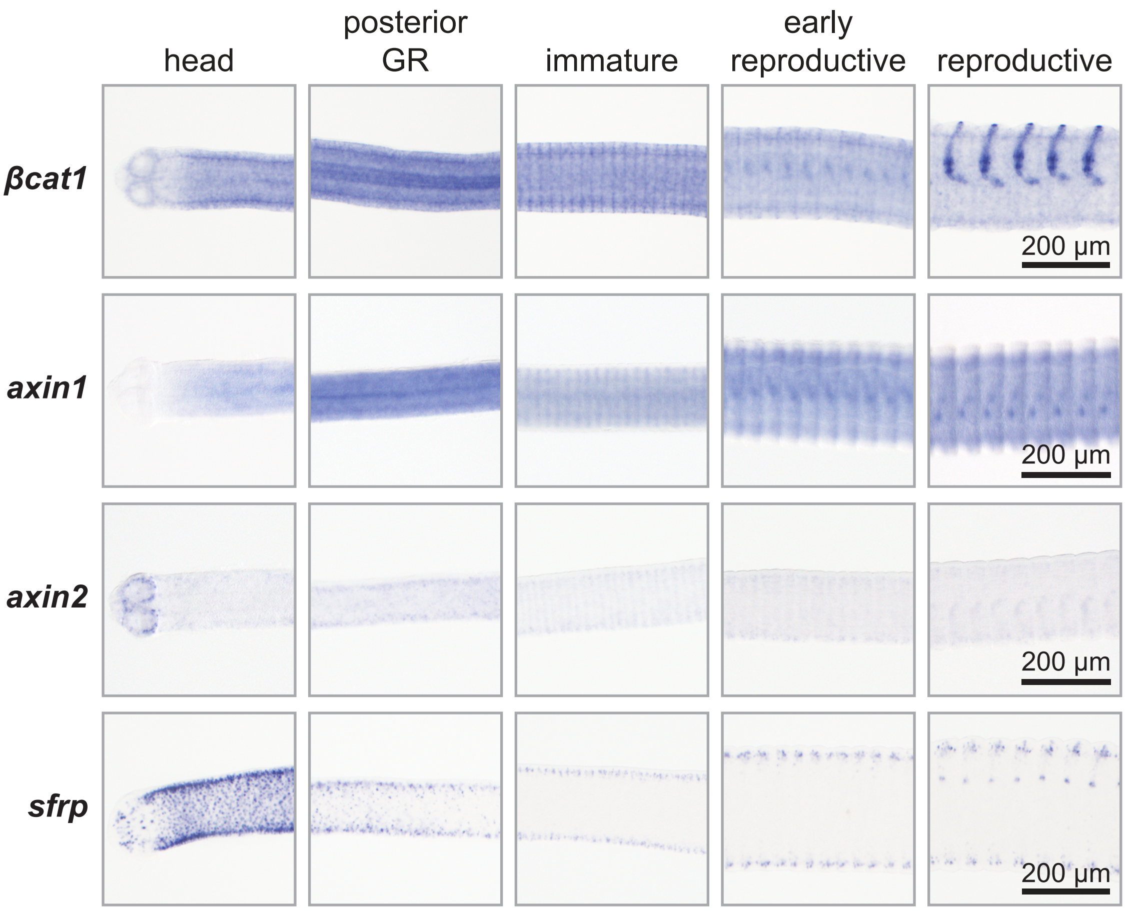

Fig. S5. WISH for *βcat1*, *axin1*, *axin2* and *sfrp*. Representative micrographs spanning different stages of development in 6-day-old worms with anterior facing left. Images for *βcat1* WISH are from the same worm displayed in Fig. 5A.

Development • Supplementary information

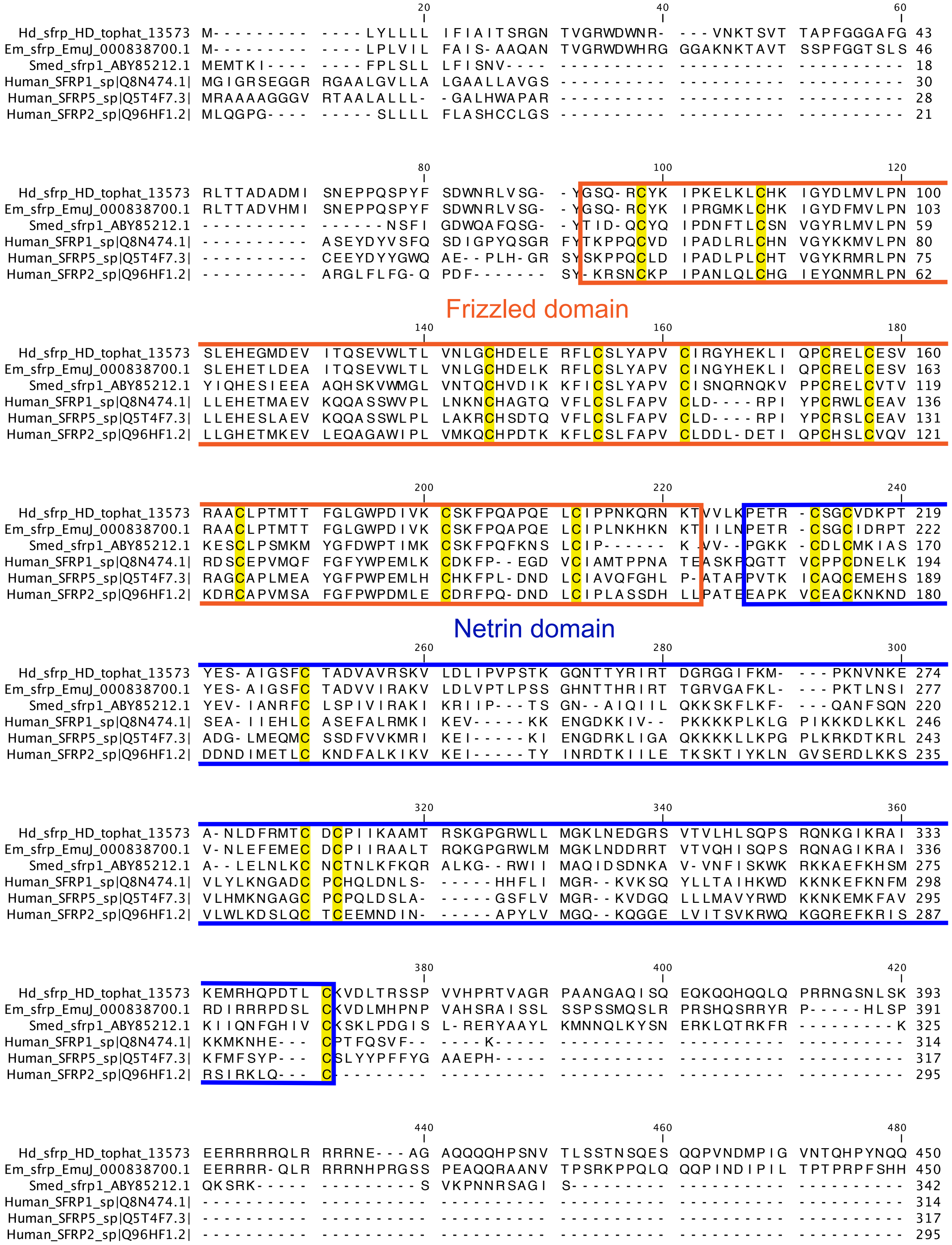

Fig. S6. Domain analysis of *H. diminuta* SFRP. BLASTp alignment of SFRPs from flatworms and human SFRP-1,-2 and -5 as references (Hd: *H. diminuta*, Em: *E. multilocularis*, Smed: *S. mediterranea*). The frizzled domain (orange) and netrin domain (blue) are marked at the most inclusive positions reported for human SFRP1 using Interpro (Blum et al., 2020). Conserved cysteines highlighted in yellow.

Development • Supplementary information

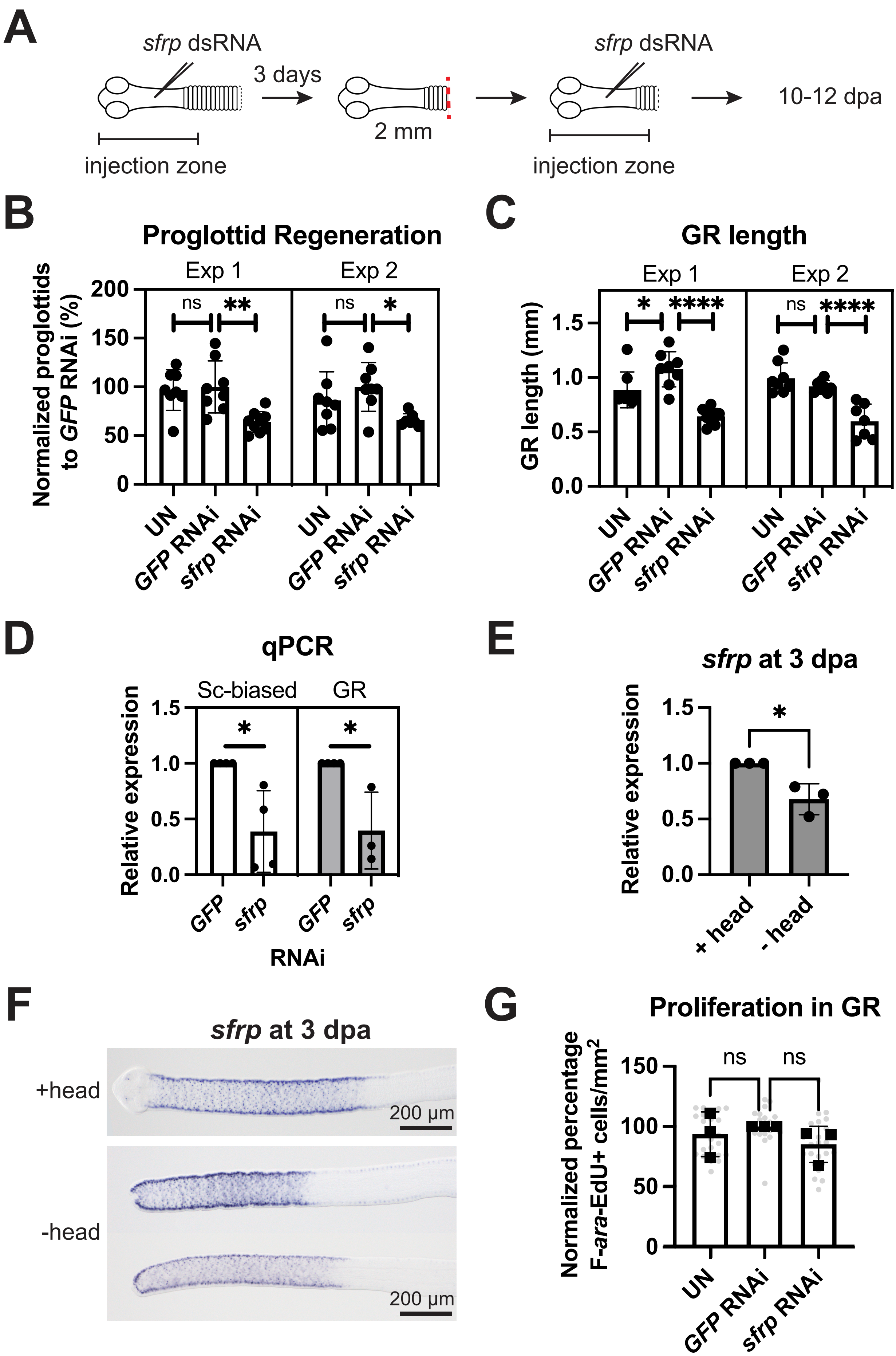

Development • Supplementary information

Fig. S7. Phenotypes observed after *sfrp* RNAi and head amputation. (A) Scheme for RNAi with dsRNA injections throughout the GR. (B-C) Quantification of proglottid regeneration (B) and GR length (C) from two independent experiments; Exp 1: n= 8, 8, 9, Exp 2: n= 8, 8, 7; one-way ANOVA with Dunnett's multiple comparison test. (D) qRT-PCR following dsRNA injection at the head/scolex (Sc-biased) or throughout the GR. Expression of *sfrp* (target) after *sfrp* RNAi compared to *GFP* RNAi (set at 1); t-test. (E) qRT-PCR for *sfrp* expression compared to +head (set at 1); t-test. (F) WISH for *sfrp* at representative worm anteriors from +/- head worms 3 dpa. Anterior facing left. (D) Quantification of F-*ara*-EdU+ cells normalized to area from 800 μm-wide cropped region posterior to the head or to the first proglottid. Combined from single and double dsRNA injections throughout the GR with significant *sfrp* knockdown. All samples were normalized to the mean of *GFP* RNAi worms set to 100%. Bars= means, gray circles= individual worms; N= 3, n= 22, 21, 23; one-way ANOVA with Dunnett's multiple comparison test. Error bars= SD.

Development • Supplementary information

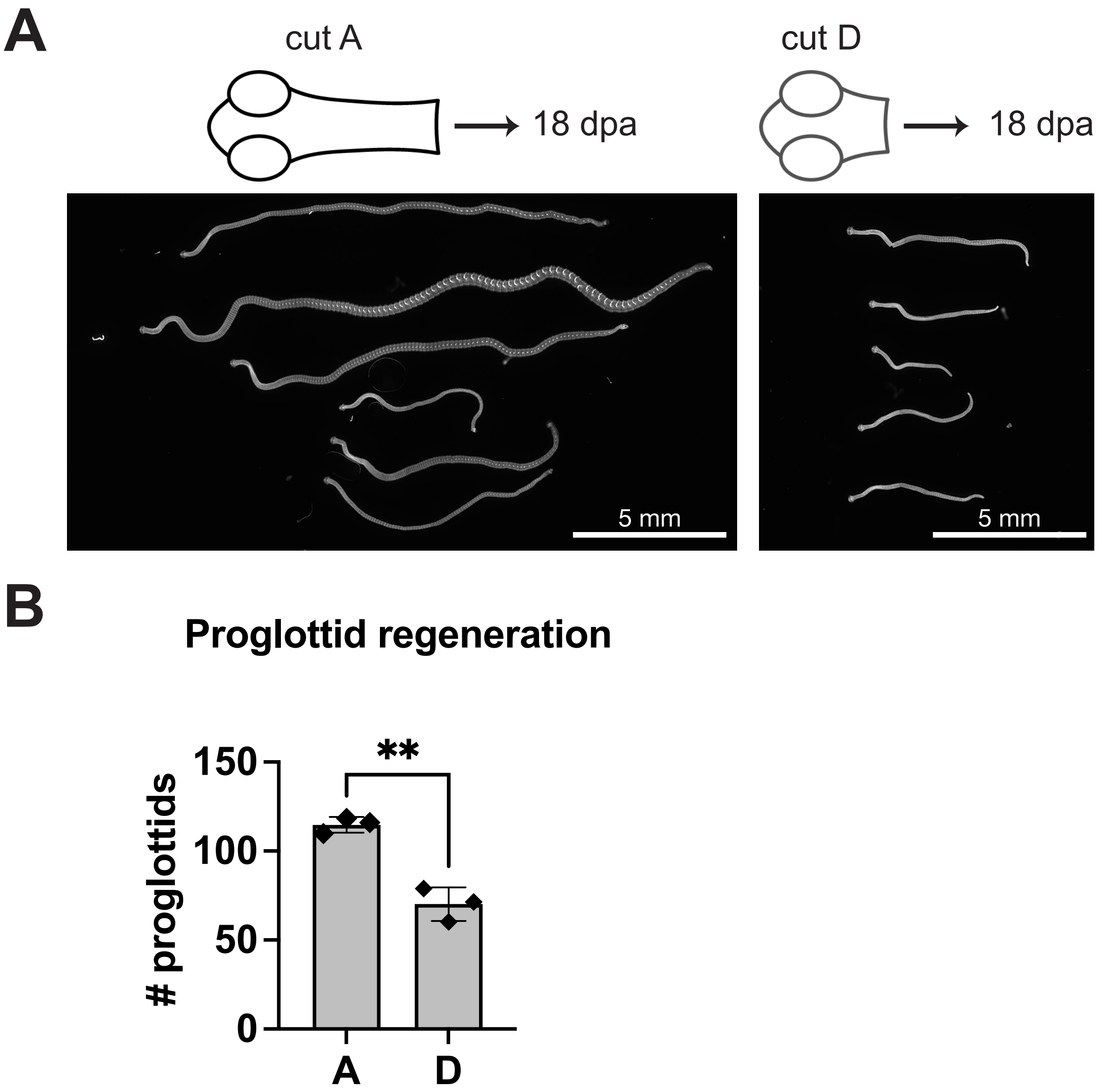

Fig. S8. Comparison of regenerates from longest vs. shortest fragment groups after re-amputation. (A) DAPI-stained regenerates at 18 dpa. (B) Proglottid regeneration at 18 dpa. N= 3, n= 20, 17; t-test; error bars= SD.

Development • Supplementary information

Table S1. *βcatenin* paralogs in *H. diminuta*.

βcat1				
Query sp.	Query Accession	Reference	Best match to *H. diminuta**	E-value
E. multilocularis	EmuJ_001007700	Montagne et al., 2019	WMSIL1_LOCUS14475	0
S. mansoni	Smp_023550	Montagne et al., 2019	WMSIL1_LOCUS14475	0
S. mediterranea	ABW79875.1	Su et al., 2017	WMSIL1_LOCUS14475	2.40E-98
βcat2				
Query sp.	Query Accession	Reference	Best match to *H. diminuta**	E-value
E. multilocularis	EmuJ_001103600	Montagne et al., 2019	WMSIL1_LOCUS880	7.20E-148
S. mansoni	Smp_173990	Montagne et al., 2019	WMSIL1_LOCUS880	5.10E-90
S. mediterranea	ABW79874.1	Su et al., 2017	WMSIL1_LOCUS880	2.80E-33
βcat3/4				
Query sp.	Query Accession	Reference	Best match to *H. diminuta**	E-value
E. multilocularis	EmuJ_000572500	Montagne et al., 2019	WMSIL1_LOCUS6332	0
S. mansoni	Smp_134000	Montagne et al., 2019	WMSIL1_LOCUS6332	4.00E-18
S. mediterranea	KY196224	Su et al., 2017	none	
S. mediterranea	KY196225	Su et al., 2017	none	

*Genome assembly PRJEB30942

Table S2. Primers and transcripts used in this study.

Available for download at

https://journals.biologists.com/dev/article-lookup/doi/10.1242/dev.204781#supplementary-data

Table S3. Source data used in this study.

Available for download at
https://journals.biologists.com/dev/article-lookup/doi/10.1242/dev.204781#supplementary-data
